# CO_2_ adsorption and activation on AuO(CO_2_)_*n*_^−/+^ (*n* = 1–3) clusters: a theoretical study

**DOI:** 10.1039/d5ra04472c

**Published:** 2025-09-30

**Authors:** Wei Huang, Wenbao Zhao, Zonghui Guo, Shihu Du, Jincheng Tian, Ruoying Zhang, Haiyan Han, Zhi Zhao, Wei Pei, Ruili Shi, Hua Xie

**Affiliations:** a School of Mathematics and Physics Science and Engineering, Hebei Computational Optical Imaging and Photoelectric Detection Technology Innovation Center, Hebei International Joint Research Center for Computational Optical Imaging and Intelligent Sensing, Hebei University of Engineering Handan 056038 China hanhy0226@163.com zhaozhi@hebeu.edu.cn shiruili@hebeu.edu.cn; b School of Chemistry and Chemical Engineering, Shandong University Jinan 250100 China; c State Key Laboratory of Molecular Reaction Dynamics, Dalian Institute of Chemical Physics, Chinese Academy of Sciences Dalian 116023 China xiehua@dicp.ac.cn; d College of Physics Science and Technology, Yangzhou University Yangzhou 225009 China

## Abstract

The geometric and electronic properties of AuO(CO_2_)_*n*_^−/+^ (*n* = 1–3) clusters have been systematically investigated using density functional theory (DFT). All anionic ground states are singlets, whereas the cationic counterparts are triplets. Anions prefer distorted CO_3_-like binding and, at *n* = 3, an oxygen-bridged ring, while cations retain near-linear CO_2_ with modest perturbation. The thermodynamics at 298 K show favorable first and second adsorption on anions and an unfavorable third step, consistent with site saturation. In cations the first step is favorable, the second weakly favorable, and the third slightly unfavorable. Natural population analysis (NPA) and Natural bond orbital (NBO) analyses indicate stronger charge acceptance and higher Au–O bond order in anions than in cations. These results identify charge state and saturation as the primary controls of bonding across this size range.

## Abstract 2

First DFT elucidation of charge- and size-dependent CO_2_ activation and binding motifs in AuO(CO_2_)_*n*_^+/−^ clusters.

## Graphical abstract

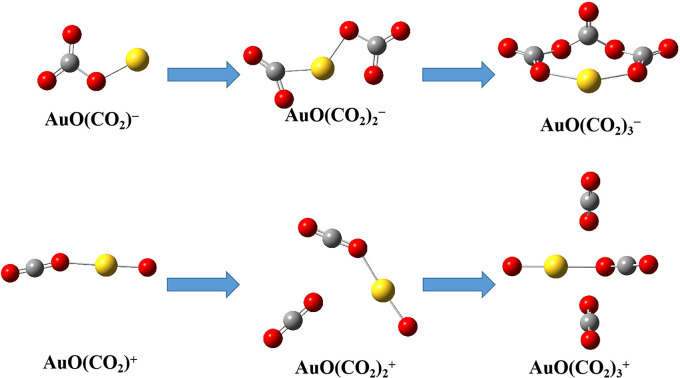

## 1 Introduction

Over the past century, the widespread use of fossil fuels has resulted in a notable elevation of carbon dioxide levels in the atmosphere, precipitating a cascade of environmental concerns, including global warming, ocean acidification, and sea level rise.^1–3^ A substantial body of research has been dedicated to the storage and fixation of carbon dioxide. Converting CO_2_ into value-added chemicals, fuels, or materials would transform it into an abundant and inexpensive carbon source. CO_2_ reduction begins with charge transfer, activating the C=O bonds and generating anionic radicals.^4^ However, CO_2_ is a highly stable molecule with strong bond energy and no dipole moment, making the reduction process challenging. Therefore, a suitable catalyst is essential to lower the activation barrier for CO_2_ reduction. A range of approaches have been widely explored for CO_2_ reduction, including electrocatalysis, biocatalysis, and photocatalysis.^5–10^

Although CO_2_ reduction is crucial for both environmental protection and chemical applications, the intrinsic mechanisms of the reaction remain poorly understood due to the complexity of the environment. Isolated gas-phase clusters with clear structural definition serve as valuable models for studying chemical reactions, enabling comprehensive exploration of molecular structures and activation mechanisms,^11–20^ while also providing valuable insights into the mechanistic steps involved in CO_2_ activation reactions.

Metal oxides, in particular, have demonstrated substantial catalytic capabilities in various chemical processes, including the activation and reduction of CO_2_. Metal oxide materials, such as TiO_2_, CuO, and CeO_2_, have been extensively used in photocatalytic and electrocatalytic applications due to their unique surface reactivity, stability, and redox properties.^21–23^ These metal oxides are known to interact with CO_2_, facilitating electron transfer and leading to the formation of carbonate or other intermediates that are crucial for CO_2_ conversion into value-added products.^24–26^ Infrared photodissociation studies reveal that ScO^+^, YO^+^, and HoO^+^ cations undergo transformation from solvated states to carbonate structures upon binding CO_2_, whereas LaO^+^ only forms solvated structures.^27–29^ Meanwhile, highly oxygenated metal oxides have also gained much attention. Liu *et al.* found that with additional CO_2_ coordination, Sc_2_O_2_^+^ and Sc_3_O_4_^+^ cations can effectively promote the fixation of CO_2_ into carbonate groups.^30,31^ Reactions of NiO_2_^+^, NbO_2_^+^, TaO_2_^+^, and TaO_3_^+^ cations with multiple CO_2_ molecules have shown no substantial CO_2_ activation.^32–35^ Through infrared spectroscopy studies on the interaction between Mn_*x*_O_*y*_^+^ (*x* = 2–5, *y* ≥ *x*) and CO_2_, Lang *et al.* revealed that the interaction is primarily electrostatic.^36^ Mikolaj *et al.* reported that CO_2_ activation on copper oxide anions primarily leads to CO_3_ formation.^37^

Meanwhile, there is a notable lack of research focused on the ability of metal oxide anions to induce CO_2_ carbonation. Hossain and co-workers observed that W_*x*_O_*y*_^−^ shows no evidence of dissociative adsorption of CO_2_.^38^ In [TiO_*x*_(CO_2_)_*y*_]^−^ systems, the study demonstrates a diversity of ligand motifs depending on the oxidation state, with carbonate ligands being the most stable across all oxidation levels, and additional oxalate, *η*^2^-(C,O), *η*^2^-(O,O), and carbonyl ligands observed at lower oxidation states.^39^

In the context of gold-based catalysts, well-defined gas-phase clusters have exhibited distinct adsorption behaviors toward small molecules such as O_2_, CO, and N_2_, often influenced by cluster size and charge state.^40–42^ Molecular oxygen can chemisorb as superoxo or peroxo species depending on the cluster size and the charge state.^40^ Molecular nitrogen, in contrast, is typically weakly adsorbed and may only be observed under cryogenic conditions.^42^ These adsorption characteristics are underpinned by the unique electronic properties of gold clusters, including relativistic effects and quantum size-dependent behavior, which also play a critical role in their catalytic performance.^43,44^ Recent reviews on gold catalysis further emphasize the significant effects of particle size and support interactions, highlighting the importance of interfacial charge transfer and dynamic redox cycles in gold-mediated transformations.^45–47^

Despite these advances, the reactivity of gold oxide clusters toward CO_2_ remains underexplored. Although earlier gas-phase studies have characterized anionic AuO *via* photoelectron spectroscopy, reporting the electron affinity of neutral AuO and its spin–orbit splitting,^48^ and the electronic structure of cationic AuO has been characterized theoretically,^49^ systematic studies on CO_2_ adsorption and activation on AuO_*n*_ clusters are still lacking.^50^ Both cationic and anionic Au_*n*_O_*m*_ clusters have demonstrated reactivity toward small molecules, suggesting their potential utility in CO_2_ conversion.^51,52^ Given the unique electronic properties of gold and its potential to enhance catalytic performance, this work is dedicated to exploring the adsorption and activation of CO_2_ on AuO(CO_2_)_*n*_^−/+^ (*n* = 1–3) clusters. By employing advanced quantum chemical methods, we systematically examine the geometric and electronic structures of these clusters, analyze their charge distributions, and elucidate the underlying mechanisms that govern CO_2_ activation. The insights gained from this study are expected not only to deepen our fundamental understanding of CO_2_ conversion processes in gold-based systems but also to provide valuable guidelines for the rational design of efficient catalysts for CO_2_ reduction.

## 2 Theoretical method

The structural searching program Molclus^53^ was employed to generate candidate initial structures for AuO(CO_2_)_*n*_^−/+^ (*n* = 1–3) clusters. Geometry optimizations were performed at the B3LYP-D3 (ref. 54–56)/def2-TZVP^57,58^ level of theory. This level is a commonly used, well-balanced choice for Au chemistry. Literature benchmarks on the def2 basis framework and on gold bonding, together with Au-oxide case studies, support its use for Au–X bonding.^57,59,60^ To assess functional and basis set sensitivity, key isomers were re-evaluated at five cross-check levels: (i) B3LYP-D3 with the LANL2DZ^61–63^ effective core potential for Au and 6-311+G(3df)^64^ for C and O; (ii) ωB97X-D^65^ with the same LANL2DZ/6-311+G(3df); (iii) M06-2X^66^ with LANL2DZ/6-311+G(3df); (iv) ωB97X-D/def2-TZVP; (v) M06-2X/def2-TZVP. The data are summarized in Tables S1 and S2 in the SI. Table S1 shows that the relative energy with zero-point energy correction remains consistent for clusters with *n* = 1 and *n* = 3 across all tested methods. For *n* = 2, some functional dependence in relative energy is observed. Though Table S2 shows that the geometric structures remain largely consistent. Harmonic frequency analysis was conducted at the same level of theory with geometry optimizations to ensure that the optimized structures corresponded to true minima on the potential energy surfaces, with no imaginary frequencies observed. Gibbs free energies at 298 K were obtained from the same frequency calculations to evaluate the thermodynamic favorability of CO_2_ adsorption. Different spin multiplicities were evaluated, and the results are summarized in Table 1. The lowest-energy states for AuO(CO_2_)_*n*_^−^ (*n* = 1–3) corresponds to singlet ground states, while the lowest-energy state for AuO(CO_2_)_*n*_^+^ (*n* = 1–3) corresponds to triplet ground states. To quantify bonding and activation we carried out single-point Natural Bond Orbital analysis (NBO)^67^ and Natural Population Analysis (NPA) on the optimized structures. All calculations were performed using the Gaussian 09 program.^68^

**Table 1:** Spin multiplicities and corresponding energies of the AuOCO_2_^−/+^ clusters

Isomer	Spin multiplicity	Energy (Hartree)
1A^−^	1	−399.711862
	3	−399.706148
	5	−399.500696
	7	−399.340932
1A^+^	1	−399.207940
	3	−399.272561
	5	−399.204922
	7	−399.047520

## 3 Result and analysis

### 3.1 Structures and relative energies

**Fig. 1:**
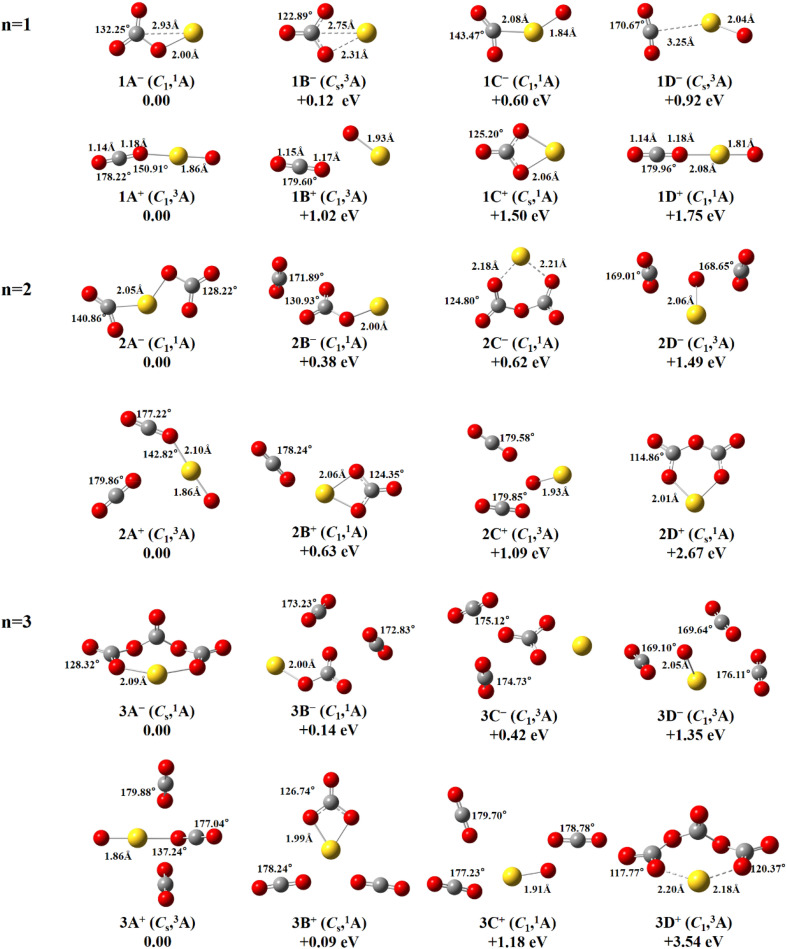
Optimized structures of the ground state and additional selected low-lying isomers of AuO(CO_2_)_*n*_^−/+^ (*n* = 1–3) calculated at the B3LYP-D3/def2-TZVP level of theory (Au, yellow; carbon, gray; oxygen, red). The symmetry, electronic state, the relative energy (eV), bond length (Å) and bond angle (in degrees) are indicated.

Fig. 1 presents the structures, symmetries, spin multiplicity, and relative energies with zero point energy (ZPE) correction for the ground state structures and selected low-lying isomers of AuO(CO_2_)_*n*_^−/+^ (*n* = 1–3) clusters. The isomers are arranged in ascending order of energy and labeled as *n*A^−/+^, *n*B^−/+^, *n*C^−/+^ and so on.

#### 3.1.1 AuO·CO_2_^−^

The lowest-energy isomer 1A^−^ (*C*_1_ symmetry, singlet state) features a significantly distorted CO_3_ fragment. The C–O bond lengths of 1.25 Å, 1.23 Å, and 1.41 Å and the O–C–O bond angles are 132.25°, 107.21° and 120.54°, resulting in an asymmetric structure. In contrast, isomer 1B^−^ (*C*_s_ symmetry, triplet state) lies 0.12 eV higher in energy than 1A^−^. 1B^−^ also contains a CO_3_ fragment like 1A^−^. The O–C–O angles are 122.89°, 122.90° and 114.21°, and the C–O bond lengths are 1.30 Å, 1.30 Å, and 1.23 Å. The geometric distortion is significantly reduced compared to 1A^−^ but remains clearly different from that of a typical carbonate ion. Isomer 1C^−^ (*C*_1_ symmetry, singlet state) is 0.63 eV higher in energy than 1A^−^. The gold atom coordinates to the carbon of CO_2_ in a monodentate fashion, slightly distorting its linear geometry. By contrast, isomer 1D^−^ (*C*_s_ symmetry, triplet state), which is 0.92 eV higher in energy than 1A^−^, features weak interaction between Au and CO_2_, allowing the CO_2_ molecule to retain its nearly linear geometry.

#### 3.1.2 AuO·CO_2_^+^

The lowest-energy isomer 1A^+^ (*C*_1_ symmetry, triplet state) adopts a nearly linear CO_2_ unit bonded to the AuO moiety *via* a distorted C–O–Au angle of 150.91°. In isomer 1B^+^ (*C*_1_ symmetry, triplet state), which lies 1.02 eV higher in energy than 1A^+^, the gold atom only forms a terminal coordination with a single oxygen atom at a bond length of 1.93 Å. Meanwhile, the nearby CO_2_ molecule retains its nearly linear geometry. This structure highlights the absence of significant perturbation to the CO_2_ molecule, as its geometry is nearly identical to that of a free CO_2_ molecule. In isomer 1C^+^ (*C*_s_ symmetry, triplet state), the gold atom lies 2.06 Å away from the two oxygen atoms of the distorted CO_3_-like unit. This structure resembles 1B^−^, but in 1C^+^ the Au–O distances to the CO_3_-like fragment are shorter. 1D^+^ (*C*_1_ symmetry, singlet state) lies 1.75 eV higher in energy than 1A^+^. The structure is characterized by a nearly linear arrangement between the AuO unit and the CO_2_ molecule, with both the O–C–O and O–Au–O bond angles approaching 180°.

#### 3.1.3 AuO(CO_2_)_2_^−^

For AuO(CO_2_)_2_^−^, in the lowest-energy isomer 2A^−^ (*C*_1_ symmetry, singlet state), the gold atom coordinates to two CO_2_ molecules. One binds *via* its carbon atom in a monodentate manner, while the other interacts terminally through an oxygen atom. This asymmetric coordination results in a non-linear spatial arrangement of the CO_2_ molecules relative to the Au center. In isomer 2B^−^ (*C*_1_ symmetry, singlet state), which lies 0.38 eV above 2A^−^, one CO_2_ molecule forms a CO_3_-like ligand with the AuO unit, similar to isomer 1A^−^, while the second CO_2_ molecule retains a nearly linear geometry with C–O bond lengths of 1.16 Å and an O–C–O bond angle of 171.89°. Positioned 0.62 eV above 2A^−^, isomer 2C^−^ (*C*_1_ symmetry, singlet state) adopts a non-planar, ring-like geometry in which the Au–O distances are 2.18 and 2.21 Å as shown in Fig. 1. In 2D^−^ (*C*_1_ symmetry, triplet state), which is 1.49 eV higher in energy than 2A^−^, both CO_2_ molecules retain geometries close to those of free CO_2_.

#### 3.1.4 AuO(CO_2_)_2_^+^

The lowest-energy isomer 2A^+^ (*C*_1_ symmetry, triplet state) retains the O–Au–CO_2_ coordination framework observed in 1A^+^ but exhibits a reduced C–O–Au bond angle. A free CO_2_ molecule is present nearby, maintaining a nearly linear geometry with an O–C–O bond angle of 179.86°. Isomer 2B^+^ (*C*_1_ symmetry, singlet state) lies 0.63 eV higher in energy than 2A^+^. Its central structure closely resembles that of 1C^+^, featuring a cyclic CO_3_-like unit bonded to the gold atom. The second CO_2_ molecule is nearly linear, with an O–C–O bond angle of 178.24°. Isomer 2C^+^ (*C*_1_ symmetry, triplet state) is 1.09 eV higher in energy than 2A^+^. The gold atom coordinates with a single oxygen atom, forming an end-on coordination bond with a length of 1.93 Å. Both CO_2_ molecules remain nearly linear, with O–C–O bond angles of 179.58° and 179.85°, respectively. Isomer 2D^+^ (*C*_s_ symmetry, singlet state) is 2.67 eV higher in energy than 2A^+^. The structure of 2D^+^ contains a cyclic CO_3_-like unit interacting with the gold atom, characterized by an Au–O bond length of 2.01 Å.

#### 3.1.5 AuO(CO_2_)_3_^−^

Ground-state isomer 3A^−^ (*C*_s_ symmetry, singlet state) forms a eight-membered ring *via* multiple bridging oxygens. This cyclic arrangement contrasts sharply with smaller clusters (*n* = 1–2), demonstrating size-dependent structural evolution. Isomer 3B^−^ (*C*_1_ symmetry, singlet state), which is 0.14 eV higher in energy than 3A^−^, the core structure resembles that of 1A^−^, while the other two CO_2_ units sit apart with O–C–O bond angles of 173.23° and 172.83°, maintaining near-linear geometries. Isomer 3C^−^ (*C*_1_ symmetry, triplet state) is 0.42 eV higher in energy than 3A^−^. The central structure of 3C^−^ resembles that of 1B^−^. And two nearly linear CO_2_ molecules are positioned nearby. In isomer 3D^−^ (*C*_1_ symmetry, triplet state) lying 1.35 eV above 3A^−^, all three CO_2_ molecules are positioned around the AuO unit. The O–C–O bond angles of 169.10°, 169.64° and 176.11° show that each CO_2_ retains a near-linear geometry.

#### 3.1.6 AuO(CO_2_)_3_^+^

The lowest-energy isomer 3A^+^ (*C*_s_ symmetry, triplet state) extends the O–Au–CO_2_ coordination framework of 1A^+^, with a further reduced C–O–Au angle of 137.24°. Two symmetrically arranged CO_2_ ligands retain near-linear geometries, demonstrating charge distribution symmetry. At just 0.09 eV above 3A^+^, isomer 3B^+^ (*C*_s_ symmetry, singlet state) contains a central cyclic CO_3_-like unit bonded to the gold atom through a 1.99 Å Au–O bond, resembling the coordination in 1C^+^. Two nearly linear CO_2_ molecules are symmetrically positioned on either side. Isomer 3C^+^ (*C*_1_ symmetry, singlet state) lies 1.18 eV above 3A^+^ in energy and contains a central gold atom bonded to a single oxygen atom at a bond length of 1.91 Å. Surrounding the central unit are three CO_2_ molecules with slightly bent geometries, displaying O–C–O bond angles of 179.70°, 178.78°, and 177.23°. Isomer 3D^+^ (*C*_1_ symmetry, triplet state), lying 3.54 eV above 3A^+^, has an overall structure similar to 3A^−^ but lacks any symmetry and features longer Au–O distances.

Comparison of anionic and cationic clusters reveals that all ground-state anionic clusters with *n* = 1–3 adopt singlet spin multiplicities, whereas the cationic counterparts consistently favor triplet states. In the anionic complexes, the additional electron density promotes the formation of distorted CO_3_-like units that strongly coordinate with Au, particularly in the lowest-energy isomers. In contrast, many cationic isomers, especially in higher-lying isomers, exhibit less perturbed, nearly linear CO_2_ geometries. Consequently, anionic clusters display more pronounced structural distortions and stronger Au–CO_3_ interactions, whereas cationic clusters tend to maintain the linear geometry of CO_2_ throughout.

### 3.2 Activation and bonding indicators

To further elucidate the evolution of structural stability in the AuO(CO_2_)_*n*_^−/+^ (*n* = 1–3) clusters, we evaluate the 298 K thermodynamics using two complementary quantities.

The total binding free energy referenced to AuO^−/+^ and *n* isolated CO_2_ molecules is*G*_bind_(*n*) = [*G*_298_(AuO^−/+^) + *n G*_298_(CO_2_)] − *G*_298_(AuO(CO_2_)_*n*_^−/+^)where a more positive value of *G*_bind_ indicates stronger stabilization relative to the separated components.

The stepwise adsorption free energy for adding CO_2_ is defined as:Δ*G*_ads_(*n*) = *G*_298_((AuO(CO_2_)_*n*_^−/+^)^min^) − *G*_298_((AuO(CO_2_)_*n*−1_^−/+^)^min^) − *G*_298_(CO_2_)

Negative values indicate thermodynamically favorable adsorption.

As summarized in Table 2, for the anions, *G*_bind_ is 86.45 kJ mol^−1^ at *n* = 1, increases to 112.19 kJ mol^−1^ at *n* = 2, and then decreases to 84.99 kJ mol^−1^ at *n* = 3. The corresponding Δ*G*_ads_ values are −86.45, −25.74, and +27.20 kJ mol^−1^, indicating pronounced site saturation that adsorption of the third CO_2_ is no longer favorable at 298 K. For the cations, *G*_bind_ is 99.94 kJ mol^−1^ at *n* = 1, increases to 104.70 kJ mol^−1^ at *n* = 2, and slightly decreases to 101.03 kJ mol^−1^ at *n* = 3. The corresponding Δ*G*_ads_ values are −99.94, −4.75, and +3.67 kJ mol^−1^. This gentle evolution is consistent with predominantly electrostatic end-on coordination that keeps CO_2_ nearly linear.

**Table 2:** Thermodynamic stability of the lowest-energy structures of the AuO(CO_2_)_*n*_^−/+^ clusters (*n* = 1–3) at 298 K

Isomer	*G*(298 K) (au)	Δ*G*_ads_ (kJ mol^−1^)	*G*_bind_ (kJ mol^−1^)
1A^−^	−399.743187	−86.449805	86.449806
2A^−^	−588.432094	−25.743017	112.192823
3A^−^	−777.100835	27.202795	84.990028
1A^+^	−399.305227	−99.944870	99.944870
2A^+^	−587.986140	−4.754778	104.699649
3A^+^	−776.663846	3.665196	101.034453

To connect these thermodynamic trends with bonding, Table 3 reports activation indicators for the coordinating CO_2_ in each lowest-energy structure. The indicators include the fragment charge Δ*q*_CO_2__ from NPA, the deviation from linearity *δθ*, the shift of the asymmetric stretch Δ*ν*_as_, and Wiberg indices from NBO.

**Table 3:** Activation and bonding indicators for the coordinating CO_2_ in the lowest-energy AuO(CO_2_)_*n*_^−/+^ structures at 298 K^a^

Isomer	CO_2_ id	Δ*q*_CO_2__ (e)	*δθ* (°)	Δ*ν*_as_ (cm^−1^)	Wiberg Au–O	Wiberg Au–C	Wiberg O_AuO_–C
1A^−^	CO_2_-a	−0.502	47.75	−1138.84	0.260	0.011	0.000
2A^−^	CO_2_-a	−0.566	51.78	−679.76	0.220	0.013	1.031
	CO_2_-b	−0.522	39.14	−510.59	0.324	0.538	0.036
3A^−^	CO_2_-a	−0.206	55.07	−641.63	0.031	0.000	0.000
	CO_2_-b	−0.397	51.69	−601.93	0.354	0.000	0.000
	CO_2_-c	−0.210	64.27	−579.13	0.036	0.000	1.288
1A^+^	CO_2_-a	+0.122	1.78	+37.91	0.052	0.003	0.000
2A^+^	CO_2_-a	+0.005	2.78	+6.97	0.004	0.000	0.000
	CO_2_-b	+0.069	0.14	+29.30	0.052	0.003	0.000
3A^+^	CO_2_-a	+0.005	0.12	+0.01	0.004	0.000	0.000
	CO_2_-b	+0.005	0.12	+11.96	0.004	0.000	0.000
	CO_2_-c	+0.069	2.96	+24.51	0.052	0.003	0.000

a Isomers are the lowest-energy ones ranked by Gibbs free energy at 298 K. Δ*q*_CO_2__ is the NPA charge on each CO_2_ unit given by the sum over its C and two O atoms. *δθ* is 180° minus the O–C–O angle, positive values mean bending. Δ*ν*_as_ is the asymmetric-stretch frequency in the cluster minus the gas-phase value 2410.09 cm^−1^ computed at the same level. Wiberg indices are from NBO. “Au–O” and “Au–C” refer to contacts between Au and the atoms of that CO_2_. “O_AuO_–C” refers to the oxygen in the AuO fragment bonded to the carbon of that CO_2_. Labels CO_2_-a, CO_2_-b, CO_2_-c identify different CO_2_ units within the same isomer.

For anions, the indicators substantiate the stability evolution. In 1A^−^ the coordinating CO_2_ accepts about 0.50*e*, deviates by 47.75° from linearity and shows a very large red shift of −1138.84 cm^−1^, with Au–O Wiberg 0.260 and Au–C essentially zero. In 2A^−^ two motifs coexist. One molecule forms an O–C linkage with the AuO oxygen, characterized by Wiberg O_AuO_–C 1.031 together with Δ*q*_CO_2__ −0.566*e*, *δθ* 51.78° and Δ*ν*_as_ −679.76 cm^−1^. The other binds through carbon to Au with Wiberg Au–C 0.538, Δ*q*_CO_2__ −0.522*e*, *δθ* 39.14° and Δ*ν*_as_ −510.59 cm^−1^, accompanied by moderate Au–O contacts. In 3A^−^ the third CO_2_ closes a eight-membered ring *via* multiple bridging oxygens. No Au–C bond is present. The three CO_2_ units still accept charge but with smaller magnitude on average, and their asymmetric stretches lie at 1768 to 1831 cm^−1^ giving red shifts of −642 to −579 cm^−1^. These signatures are weaker than in 1A^−^ and match the onset of site saturation and the positive Δ*G*_ads_ for the third CO_2_ adsorption.

Cationic clusters show weak end-on electrostatic coordination. In 1A^+^ the CO_2_ fragment is nearly linear, *δθ* 1.8°. Δ*q*_CO_2__ + 0.12*e* and Δ*ν*_as_ + 38 cm^−1^. Wiberg Au–O about 0.05 and Au–C essentially zero, which indicates Au–O-dominated contact. In 2A^+^ both CO_2_ molecules remain almost linear, *δθ* up to 2.8°. Δ*q*_CO_2__ values + 0.005 and +0.069*e*, Δ*ν*_as_ values + 7 and +29 cm^−1^. Wiberg Au–O is 0.004 for unit *a* and 0.052 for unit *b*, while Au–C is negligible. In 3A^+^ three nearly linear CO_2_ units are retained. Δ*ν*_as_ spans 0 to 25 cm^−1^. Only unit *c* shows an appreciable Au–O contact with Wiberg 0.052. These indicators agree with the gentle thermodynamic evolution in Table 2, where Δ*G*_ads_ changes from −99.94 to −4.75 to +3.67 kJ mol^−1^, and they explain the persistence of linear CO_2_ in the cationic structures.

Taken together, the activation indicators give a consistent picture. In the anionic clusters, the coordinating CO_2_ shows pronounced bending and strong red shifts, together with substantial charge uptake and non-negligible Au–O and Au–C Wiberg bond orders. These signatures weaken at *n* = 3, in line with the onset of site saturation. In the cationic clusters, CO_2_ remains nearly linear with small or positive Δ*ν*_as_, minimal charge transfer, and very low Au–O bond orders. Hence, the charge state governs the activation strength, and increasing ligand number attenuates all indicators.

## 4 Conclusion

This study systematically investigates the geometric and electronic properties of AuO(CO_2_)_*n*_^−/+^ (*n* = 1–3) clusters using density functional theory. The analysis of various isomers reveals that the coordination of the AuO unit with CO_2_ induces significant structural distortions and diverse binding motifs, which vary with both the charge state and the cluster size. For the anionic clusters, strong Au coordination results in pronounced distortions of the CO_2_ moiety, leading to CO_3_-like configurations together with larger charge acceptance on the coordinating CO_2_ and enhanced Au–O bonding signatures. Thermodynamic analysis at 298 K, adsorption of the first two ligands is favorable, whereas the third ligand becomes unfavorable, indicating clear site saturation. In contrast, the cationic clusters exhibit more localized charge distributions, with the CO_2_ molecules largely retaining their near-linear geometries and experiencing only minor perturbations upon coordination. The thermodynamic evolution with increasing ligand number is gentle for cations, and the third adsorption is slightly unfavorable at 298 K. NPA and NBO analyses corroborate this picture by showing modest charge transfer and low Au–O bond orders for cations, in contrast to stronger charge acceptance and higher Au–O bond orders for anions. Size-dependent structural evolution is evident, with ring-closure motifs emerging at *n* = 3. These findings deepen the mechanistic understanding of CO_2_ activation on gold–oxide clusters and provide guidance for designing Au-based motifs in which charge state and site saturation jointly govern adsorption thermodynamics and bonding.

## Conflicts of interest

The authors declare that they have no known competing financial interests or personal relationships that could have appeared to influence the work reported in this paper.

## Supplementary Material

RA-015-D5RA04472C-s001

## Data Availability

The data supporting this article have been included as part of the supplementary information (SI). Supplementary information: (i) Table S1 summarizing method sensitivity for AuO(CO_2_)_*n*_^−/+^ (*n* = 1–3); (ii) Table S2 reporting percent differences in Au–O bond lengths and O–C–O angles for each method combination relative to B3LYP/def2-TZVP; and (iii) optimized Cartesian coordinates of the low-lying AuO(CO_2_)_*n*_^−/+^ isomers at the B3LYP-D3/def2-TZVP level. See DOI: https://doi.org/10.1039/d5ra04472c.
