# Supplementary material for: CO2 adsorption and activation on AuO(CO2)n−/+ (n = 1–3) clusters: a theoretical study
Source: RSC Adv. 2025 Sep 30;15(43):36084–92. doi: 10.1039/d5ra04472c (PMC12481190; doi:10.1039/d5ra04472c)
Supplement: RA-015-D5RA04472C-s001 [file RA-015-D5RA04472C-s001.pdf]

Supplementary Information (SI) for RSC Advances.
This journal is © The Royal Society of Chemistry 2025

CO_2 Adsorption and Activation on $AuO(CO_2)_n^{-/+}$ ($n = 1 - 3$) Clusters: A Theoretical Study

Wei Huang,[a] Wenbao Zhao,[a] Zonghui Guo,[a] Shihu Du,[b,c] Jincheng Tian,[a] Ruoying Zhang,[a] Haiyan Han,[a*] Zhi Zhao,[a,c*] Wei Pei,[d] Ruili Shi,[a*] Hua Xie[c*]

[a]*School of Mathematics and Physics Science and Engineering, Hebei Computational Optical Imaging and Photoelectric Detection Technology Innovation Center, Hebei International Joint Research Center for Computational Optical Imaging and Intelligent Sensing, Hebei University of Engineering, Handan 056038, China*

[b]*School of Chemistry and Chemical Engineering, Shandong University, Jinan 250100, China*

[c]*State Key Laboratory of Molecular Reaction Dynamics, Dalian Institute of Chemical Physics, Chinese Academy of Sciences, Dalian 116023, China*

[d]*College of Physics Science and Technology, Yangzhou University, Yangzhou 225009, China*

*Corresponding authors: hanhy0226@163.com; zhaozhi@hebeu.edu.cn; shiruili@hebeu.edu.cn; xiehua@dicp.ac.cn

Table S1 Method sensitivity summary for $AuO(CO_2)_n^{-/+}$ (n = 1 - 3): ZPE-corrected relative energies across six methods.

Isomer	Relative Energy (eV)					
	comb1	comb2	comb3	comb4	comb5	comb6
$1A^-$	0.00	0.00	0.00	0.00	0.00	0.00
$1B^-$	0.12	0.19	0.13	0.13	0.09	0.10
$1C^-$	0.60	0.79	1.06	1.23	0.64	0.87
$1D^-$	0.92	1.30	1.39	1.32	1.31	1.35
$2A^-$	0.00	0.00	0.00	0.00	0.00	0.00
$2B^-$	0.38	0.24	0.27	0.14	0.57	0.41
$2C^-$	0.62	0.52	-0.02	-0.13	0.47	0.31
$2D^-$	1.49	1.38	1.62	1.44	1.74	1.74
$3A^-$	0.00	0.00	0.00	0.00	0.00	0.00
$3B^-$	0.14	0.11	0.84	0.85	0.77	0.58
$3C^-$	0.42	0.40	1.22	1.22	0.86	0.89
$3D^-$	1.35	1.35	2.22	2.29	1.81	1.91
$1A^+$	0.00	0.00	0.00	0.00	0.00	0.00
$1B^+$	1.02	0.97	0.19	0.14	0.99	0.98
$1C^+$	1.50	1.44	1.74	1.66	1.64	1.56
$1D^+$	1.75	1.77	1.92	1.88	1.81	1.86
$2A^+$	0.00	0.00	0.00	0.00	0.00	0.00
$2B^+$	0.63	0.61	0.81	0.87	0.69	0.64
$2C^+$	1.09	1.01	0.06	0.05	0.00	0.03
$2D^+$	2.67	2.58	3.06	3.07	3.21	1.53
$3A^+$	0.00	0.00	0.00	0.00	0.00	0.00
$3B^+$	0.09	0.11	0.13	0.23	0.04	0.04
$3C^+$	1.18	1.16	0.98	0.98	1.42	1.16
$3D^+$	3.54	3.43	2.85	2.78	3.38	3.74

Methods:

comb1: B3LYP-D3/Def2-TZVP

comb2: B3LYP-D3/LANL2DZ/6-311+G(3df)

comb3: M06-2X /Def2-TZVP

comb4: M06-2X/LANL2DZ/6-311+G(3df)

comb5: ωB97X-D/ Def2-TZVP

comb6: ωB97X-D /LANL2DZ/6-311+G(3df)

Table S2 Percent differences of Au-O bond length and O-C-O angle for each method combination relative to B3LYP/def2-TZVP

Isomer	percent change in Au-O bond length P_{bond}					percent change in O-C-O angle P_{angle}				
	comb1	comb2	comb3	comb4	comb5	comb1	comb2	comb3	comb4	comb5
$1A^-$	0.50%	-0.50%	-0.50%	-1.50%	-1.00%	-0.20%	-0.74%	-0.68%	-0.08%	-0.33%
$1B^-$	2.16%	-3.90%	-1.73%	-2.60%	-0.43%	-0.29%	1.05%	0.92%	0.71%	0.45%
$1C^-$	1.09%	-0.54%	0.54%	-1.09%	0.00%	0.30%	1.14%	2.11%	0.02%	0.45%
$1D^-$	0.98%	1.47%	2.45%	-0.49%	0.98%	-0.53%	1.21%	1.03%	1.87%	1.87%
$2A^-$	1.98%	-0.99%	-0.50%	-0.99%	-0.50%	0.48%	1.82%	2.60%	0.16%	0.72%
$2B^-$	0.81%	-0.24%	-0.11%	-1.25%	-0.61%	-0.02%	-0.66%	-0.50%	-0.30%	-0.19%
$2C^-$	1.07%	0.15%	0.28%	-0.90%	-0.51%	-0.25%	1.12%	1.08%	0.31%	0.26%
$2D^-$	1.16%	3.14%	4.23%	0.22%	1.33%	0.71%	0.98%	1.50%	0.73%	1.08%
$3A^-$	0.00%	0.96%	1.44%	-0.48%	0.00%	-0.05%	0.92%	0.87%	0.03%	0.05%
$3B^-$	0.67%	-0.01%	0.13%	-1.18%	-0.89%	0.08%	-0.69%	-0.56%	-0.50%	-0.34%
$3C^-$	2.25%	-3.80%	-2.00%	-2.44%	-0.44%	0.02%	-0.33%	-0.24%	-0.15%	-0.09%
$3D^-$	1.39%	3.65%	4.13%	-0.30%	1.69%	0.34%	0.62%	0.91%	0.67%	0.58%
$1A^+$	0.69%	1.99%	3.69%	-0.14%	0.82%	0.29%	0.22%	0.95%	0.03%	0.98%
$1B^+$	0.55%	-1.80%	-0.19%	-3.85%	2.26%	-0.01%	-0.54%	0.22%	-0.67%	0.16%
$1C^+$	0.73%	-3.43%	-2.13%	-2.04%	-1.05%	-0.23%	1.87%	1.51%	0.95%	0.62%
$1D^+$	0.67%	-0.16%	1.05%	-0.77%	0.10%	0.02%	-0.39%	0.02%	-0.57%	0.02%
$2A^+$	0.87%	1.88%	3.81%	-0.23%	1.14%	-0.01%	0.14%	0.18%	0.02%	0.04%
$2B^+$	0.86%	-3.45%	-2.30%	-2.30%	-1.17%	-0.07%	0.20%	0.21%	0.05%	0.00%
$2C^+$	0.72%	-1.64%	1.13%	-3.94%	-2.51%	0.00%	-0.28%	-0.22%	-0.63%	0.15%
$2D^+$	0.59%	-3.66%	-2.64%	-2.33%	-1.63%	-0.22%	1.15%	0.78%	0.23%	-0.03%
$3A^+$	0.93%	1.72%	3.66%	-0.25%	1.15%	0.93%	0.09%	0.14%	-0.01%	0.04%
$3B^+$	0.82%	-2.72%	-1.71%	-1.95%	-1.05%	-0.03%	0.08%	0.10%	-0.03%	-0.06%
$3C^+$	1.46%	8.41%	8.86%	4.43%	5.21%	1.46%	0.00%	-0.03%	-0.17%	-0.19%
$3D^+$	0.46%	7.91%	8.91%	3.05%	5.01%	0.46%	3.23%	3.29%	1.06%	2.29%

Methods:

comb1: B3LYP-D3/LANL2DZ/6-311+G(3df)

comb2: M06-2X /Def2-TZVP

comb3: M06-2X/LANL2DZ/6-311+G(3df)

comb4: ωB97X-D/ Def2-TZVP

comb5: ωB97X-D /LANL2DZ/6-311+G(3df)

$P_{bond} = [r_{comb\ n} - r_{ref}] / r_{ref}$

Where $r_{comb\ n}$ is the internal Au–O bond length of the AuO unit from the optimized structure at method combination comb n, and r_{ref} is the B3LYP-D3/def2-TZVP value. Positive means longer than the reference.

$P_{angle} = [\theta_{comb\ n} - \theta_{ref}] / \theta_{ref}$

Where $\theta_{comb\ n}$ is the O–C–O angle of the coordinated CO_2 from the optimized structure at method combination comb *n*, and θ_{ref} is the B3LYP-D3/def2-TZVP value. Positive means longer than the reference.

The coordinates of the low-lying $AuO(CO_2)_n^{-/+}$ isomers, optimized at the B3LYP-D3 level with Def2-TZVP, are as follows:

$1A^-$

C	-2.13820000	0.09626500	-0.00001000
O	-1.95147600	1.31428700	-0.00000300
O	-3.17825800	-0.59309300	-0.00000200
O	-1.04707200	-0.79526400	-0.00000800
Au	0.78789400	0.00019000	0.00000200

$1B^-$

O	-1.29528100	-1.09524400	-0.00006700
Au	0.74229200	0.00000000	0.00002800
C	-2.00379000	-0.00000700	-0.00006600
O	-1.29543400	1.09533000	-0.00006700
O	-3.23657500	-0.00008200	-0.00009500

$1C^-$

C	0.29372100	-1.69979200	0.00000000
O	1.48809800	-1.37425500	0.00000000
O	-0.44594700	-2.63888500	0.00000000
O	-1.26244100	1.70537600	0.00000000
Au	0.00000000	0.36279600	0.00000000

$1D^-$

O	2.02376200	-0.00002600	1.23320100
Au	0.55813000	0.00001500	-0.18034400
C	-2.67268500	-0.00003500	0.18506700
O	-2.76523100	-1.16052800	0.20441300
O	-2.76554700	1.16043200	0.20448500

$2A^-$

C	-2.36479300	0.18693600	-0.00029500
O	-1.84079300	-1.00703000	0.00035400
O	-3.54363900	0.49235200	-0.00026900
C	2.18195200	0.30854500	-0.00002900
O	2.16542400	-0.95478800	-0.00046600
O	2.94714300	1.22416600	0.00018300
O	-1.39032500	1.14442400	-0.00047800
Au	0.18221000	-0.12868200	0.00009300

$2B^-$

C	1.20857400	0.69852100	0.00019400
O	2.00468700	1.65860400	0.00084900

O	1.41112500	-0.52388200	-0.00077300
C	4.06461100	-0.47278700	-0.00008800
O	4.14460600	-0.49107200	1.15892700
O	4.14529600	-0.48854500	-1.15909400
O	-0.09683300	1.19761200	0.00066800
Au	-1.57607800	-0.15412800	-0.00006700

$2C^{-}$

C	1.47538900	1.36678200	-0.03055300
O	0.28740000	1.68578800	-0.39737300
O	2.26839700	2.06878400	0.55940100
C	1.70421200	-1.17820600	-0.00559400
O	2.56263900	-2.01236200	-0.21659000
O	1.97369600	0.10451500	-0.48245100
O	0.58640100	-1.40717500	0.60315900
Au	-1.01906200	-0.05883400	-0.00395300

$2D^{-}$

O	0.93667900	-0.00028800	-0.00000100
C	1.80641100	2.27124800	-0.00000100
O	1.85376700	2.37625900	-1.15898500
O	1.85376600	2.37625800	1.15898400
Au	-1.11966900	0.00038100	0.00000000
C	1.80478400	-2.27249500	0.00000000
O	1.85206300	-2.37752800	1.15898500
O	1.85205600	-2.37753200	-1.15898500

$3A^{-}$

C	-0.00015700	1.65482500	0.23987300
O	-0.00056800	1.75833500	1.43661900
O	1.11104400	1.64971800	-0.53924100
C	2.26675300	0.82014200	-0.18855300
O	2.01702900	-0.42185400	0.02674500
O	3.33353800	1.36756800	-0.23317500
O	-2.01707600	-0.42200700	0.02665900
Au	0.00002600	-0.95391200	0.01607000
C	-2.26674400	0.81986200	-0.18934900
O	-1.11078400	1.64925100	-0.54000100
O	-3.33333200	1.36774700	-0.23277300

$3B^{-}$

C	3.85620500	-0.51912000	-0.12555700
O	3.79181200	0.16947500	0.80865300
O	4.06372700	-1.18403700	-1.05363900

C	1.43942600	2.20489500	0.17543000
O	2.06718400	2.46346300	-0.76708300
O	0.81667300	2.08022200	1.14707600
O	-0.47118200	-1.38438000	0.53573500
Au	-1.78539600	-0.01232400	-0.09972200
C	0.86009800	-1.21335700	0.17929800
O	1.19679600	-0.26790200	-0.55798300
O	1.54898400	-2.10945500	0.70011900

$3C^{-}$

C	-3.76106600	-1.52406600	-0.24692700
O	-3.05467700	-2.05189900	-0.99971000
O	-4.54272300	-1.04718300	0.46718000
C	-0.55482900	-0.23850000	0.70426900
O	-1.73142800	-0.32231000	1.07626800
O	0.37849300	-1.04952200	1.10942500
O	-0.14767100	0.67936300	-0.12803200
C	-2.79166200	1.94323300	-0.23886200
O	-3.11659800	1.30301100	-1.15178800
O	-2.53338300	2.65808300	0.63675000
Au	2.03328200	-0.03089000	-0.11888100

$3D^{-}$

C	2.99979500	0.14535500	0.68555300
O	3.25493000	0.43396000	-0.40988800
O	2.82007200	-0.12833200	1.79821500
C	0.72752800	2.58057700	-0.45005300
O	0.36168000	2.75821800	-1.53934300
O	1.22323600	2.56429400	0.60412400
O	-0.89664800	0.78687700	0.02461100
C	-3.26189000	0.71817500	0.62554200
O	-3.65534800	0.70626600	-0.47017300
O	-3.08254200	0.73699200	1.77592200
Au	-0.03791900	-1.05735200	-0.24600000

$1A^+$

O	2.33511400	-0.29490200	-0.00001200
Au	0.50475000	0.02185100	0.00000200
C	-2.67368300	0.01108200	-0.00000400
O	-1.55398700	0.39351500	-0.00000900
O	-3.76026900	-0.32270400	0.00000100

$1B^+$

O	-0.45410800	1.24182300	-0.00023200
Au	0.96130300	-0.06911900	0.00004300
C	-3.29515400	-0.19835100	-0.00005000
O	-4.41159600	0.06875600	0.00043000
O	-2.15579800	-0.47926200	-0.00058400

$1C^+$

C	-1.84396800	0.00006500	-0.00014200
O	-1.08664100	-1.07413100	0.00012300
O	-3.03979100	0.00001000	0.00006100
O	-1.08651200	1.07411900	0.00010600
Au	0.66794100	-0.00000500	-0.00001900

$1D^+$

C	-0.00348100	-2.73735800	0.00000000
O	-0.01227200	-1.55651300	0.00000000
O	0.00420100	-3.87374800	0.00000000
O	0.01068200	2.33083400	0.00000000
Au	0.00000000	0.52176700	0.00000000

$2A^+$

O	2.03550400	-1.57900500	-0.00145800
C	-2.95217800	-1.33467800	0.00000700
O	-3.83555300	-2.06850300	0.00076800
O	-2.04957200	-0.58845700	-0.00055800
Au	0.96081400	-0.06553500	0.00008800
C	-1.26864600	2.12703100	0.00007400
O	-2.31251600	2.57776800	-0.00189200
O	-0.16028200	1.71108800	0.00221000

$2B^+$

C	2.32849000	0.60139300	0.02787200
O	3.42712900	1.05983800	0.04071100
O	1.22415800	1.30978200	-0.16594600
C	-3.07854100	0.46300900	0.00570200

O	-2.11888500	-0.19353200	-0.21805500
O	-4.01984100	1.06958000	0.20436600
O	1.97539200	-0.65898500	0.19653100
Au	0.00755300	-0.34278300	-0.00838400

$2C^{+}$

O	-0.00375300	0.13327700	1.29327800
C	-2.73762500	-1.62877600	-0.10197400
O	-3.69347400	-2.24221100	0.07292500
O	-1.76405000	-1.00337600	-0.28325600
Au	1.32526100	-0.13890600	-0.07643500
C	-2.03343400	2.07785000	-0.09122100
O	-0.86375800	2.06674100	-0.06770900
O	-3.18362600	2.08045500	-0.11554400

$2D^{+}$

C	-1.68852800	1.22734500	-0.00014400
O	-0.38128900	1.41780500	-0.00021800
O	-2.33986500	2.22481400	-0.00035000
C	-1.68853000	-1.22735900	-0.00076600
O	-2.33993300	-2.22477400	0.00003400
O	-2.26646400	0.00001900	0.00006100
O	-0.38130500	-1.41780000	-0.00029000
Au	1.03712900	-0.00000500	0.00014600

$3A^{+}$

C	1.97662600	-2.57085400	-0.80089400
O	1.33207500	-1.76880500	-0.24401000
O	2.60960800	-3.35754700	-1.35004200
C	1.97703600	2.57054600	-0.80094500
O	2.61032300	3.35695100	-1.35015400
O	1.33217100	1.76879000	-0.24400400
O	-2.36326200	0.00033600	-1.40554100
C	1.18976700	-0.00002000	1.99301800
O	0.03194300	-0.00011500	1.74748100
O	2.28887100	0.00007000	2.28610500
Au	-1.18473900	0.00005700	0.02701600

$3B^{+}$

C	2.63693500	-1.72728900	0.00011900
O	1.46173900	-1.59436800	-0.00003500
O	3.76462400	-1.89002200	-0.00032000
C	-2.60825700	-1.76884900	-0.00010000
O	-1.43508900	-1.61898900	0.00019300

O	-3.73348400	-1.94788800	-0.00022500
O	1.06078400	1.74524000	-0.00000700
Au	-0.00004100	0.05851200	0.00003000
C	-0.02205900	2.53864300	0.00002500
O	-1.09062300	1.72610300	0.00003000
O	-0.03251200	3.72023400	0.00003000

$3C^{+}$

C	-2.83845700	-1.23955300	-0.09360900
O	-1.70410900	-1.44891100	-0.34193600
O	-3.94750000	-1.08455300	0.12111400
C	3.83105600	1.08810900	-0.15369200
O	2.65650800	1.13258300	-0.37681800
O	4.94948500	1.06593100	0.04515200
O	2.02035200	-0.23682400	0.52659900
Au	0.22628600	-0.65173800	0.01613700
C	-2.53290200	2.59477700	0.01937700
O	-2.13283300	1.49912100	-0.04870900
O	-2.92125300	3.67606900	0.08619200

$3D^{+}$

C	-0.21808600	1.79588100	0.46919300
O	-0.05518100	1.62530700	1.61832400
O	0.75135300	1.94825100	-0.49256800
C	1.92893500	1.28064300	-0.30381300
O	1.98468100	0.05943600	-0.10151400
O	2.97785000	1.94902800	-0.42925600
O	-1.90318200	-0.34077300	0.00920500
Au	0.15477400	-1.12754500	0.01356400
C	-2.23753400	0.83236500	-0.20718900
O	-1.44662500	1.90977600	-0.16458100
O	-3.44227800	1.05181400	-0.54219900
